# Meig’s Velpeau’s Midwifery

**Published:** 1852-07

**Authors:** 


*A complete Treatise on Midwifery, or the Theory and Practice of Tokology, including the diseases of pregnancy, labor, and the puerperal state.* By ALF. A. L. M. VELPEAU, M. D.; translated from the French, by CHAS. D. MEIGS, M. D, Member of the American Philosophical Society, Professor of Midwifery in the Jefferson Medical College, etc.; fourth American, with the Additions from the last French edition, by WM. BYRD PAGE, Lecturer on Obstetrics in the Philadelphia Medical Institute, Consulting Surgeon to the Philadelphia Hospital, Blockley, Member of the College of Physicians, etc. With numerous illustrations. P. 652. Philadelphia; Lindsay and Blakiston; 1852. From the publishers, through S. C. Griggs & Co.

THIS is a new edition of the clearest and most systematic treatise on the subject that has fallen under our notice. The former translation of the work, was, like every thing that comes from the pen of its great author, a masterpiece of workmanship; but owing to the rapid advancement that has been made in this department of the profession, it had fallen behind the times. We are now glad to welcome a new edition of it, revised and extended by the author, with notes by the translator.

We confess we do not exactly understand the part Dr. Page has been playing in the production of this edition of the work. If all that he has done was to compare the new with other editions of the work, we see but little more propriety in the insertion of his name on the title page, with the significant *by* prefixed, than to have put first the author's, then the translator's, then the proof reader's, then the compositor's, then the pressman's, and last the binder's names; so that all should have their share of the credit of its production, be it never so small, and also enjoy the blessed privilege of displaying their respective titles and of seeing their names in print.

The mechanical execution of the work is in the publisher's best style, and would do credit to any establishment in the country. We fear, however, that small type are becoming too fashionable, for the good of the eyes of the studious. E.

